# A risk-predictive model for obstructive sleep apnea in patients with chronic obstructive pulmonary disease

**DOI:** 10.3389/fnins.2023.1146424

**Published:** 2023-03-17

**Authors:** Tianfeng Peng, Shan Yuan, Wenjing Wang, Zhuanyun Li, Ayshat Mussa Jumbe, Yaling Yu, Zhenghao Hu, Ruijie Niu, Xiaorong Wang, Jinnong Zhang

**Affiliations:** ^1^Department of Emergency Medicine, Tongji Medical College, Union Hospital, Huazhong University of Science and Technology, Wuhan, China; ^2^Department of Emergency Medicine, Renmin Hospital of Wuhan University, Wuhan, China; ^3^Department of Critical Care Medicine, Henan Provincial People's Hospital, Zhengzhou, China; ^4^Department of Critical Care Medicine, Henan University People's Hospital, Zhengzhou, China; ^5^Henan Key Laboratory for Critical Care Medicine, Zhengzhou, China; ^6^Department of Critical Care Medicine, Zhengzhou University People's Hospital, Zhengzhou, China; ^7^Department of Respiratory and Critical Care Medicine, Tongji Medical College, Union Hospital, Huazhong University of Science and Technology, Wuhan, China

**Keywords:** sleep apnea syndrome, chronic obstructive pulmonary disease, overlap syndrome, nomogram, risk factors

## Abstract

**Background:**

Obstructive sleep apnea syndrome (OSA) is increasingly reported in patients with chronic obstructive pulmonary disease (COPD). Our research aimed to analyze the clinical characteristics of patients with overlap syndrome (OS) and develop a nomogram for predicting OSA in patients with COPD.

**Methods:**

We retroactively collected data on 330 patients with COPD treated at Wuhan Union Hospital (Wuhan, China) from March 2017 to March 2022. Multivariate logistic regression was used to select predictors applied to develop a simple nomogram. The area under the receiver operating characteristic curve (AUC), calibration curves, and decision curve analysis (DCA) were used to assess the value of the model.

**Results:**

A total of 330 consecutive patients with COPD were enrolled in this study, with 96 patients (29.1%) confirmed with OSA. Patients were randomly divided into the training group (70%, *n* = 230) and the validation group (30%, *n* = 100). Age [odds ratio (OR): 1.062, 1.003–1.124], type 2 diabetes (OR: 3.166, 1.263–7.939), neck circumference (NC) (OR: 1.370, 1.098–1,709), modified Medical Research Council (mMRC) dyspnea scale (OR: 0.503, 0.325–0.777), Sleep Apnea Clinical Score (SACS) (OR: 1.083, 1.004–1.168), and C-reactive protein (CRP) (OR: 0.977, 0.962–0.993) were identified as valuable predictors used for developing a nomogram. The prediction model performed good discrimination [AUC: 0.928, 95% confidence interval (CI): 0.873–0.984] and calibration in the validation group. The DCA showed excellent clinical practicability.

**Conclusion:**

We established a concise and practical nomogram that will benefit the advanced diagnosis of OSA in patients with COPD.

## Introduction

Chronic obstructive pulmonary disease (COPD) combined with obstructive sleep apnea (OSA) is called overlap syndrome (OS), which was first proposed by Flenley in 1985 (Buist et al., 2007). OS is a disease with a high prevalence ranging from 2.9% to 65.9% (Shawon et al., 2017), with reduced diagnoses, mainly due to the lack of attention of patients and doctors and the limitation of screening tools, especially in underdeveloped areas. There are considerable differences in epidemiology, treatment, and prognosis between patients with COPD alone and patients with OS. Compared with patients with COPD alone, patients with OS have been reported to have a higher risk of cardiovascular disease, increased rate of COPD exacerbation, hospitalization, mortality, and medical costs (Hong et al., 2020; Tang et al., 2021; Zhang et al., 2022). Fortunately, studies suggest that treatment with positive airway pressure therapy significantly reduced these risks and improved patients' prognosis (Marin et al., 2010; Suri and Suri, 2021; Sterling et al., 2022). Therefore, early diagnosis and the use of non-invasive positive pressure ventilation (NPPV) are beneficial to the treatment and prognosis of patients with OS.

The gold standard in the diagnosis of OSA is polysomnography (PSG), but the lack of a large-scale laboratory in developing areas and the related costs have led to a delay in the diagnosis. The well-designed questionnaires such as Sleep Apnea Clinical Score (SACS) and modified Epworth Sleepiness Scale (mESS) have been applied as an alternative method to diagnose OSA in the absence of PSG, but they were subjective and prone to bias as revealed by a meta-analysis (Chiu et al., 2017).

Therefore, there is an imperative need for a simple and reliable method to identify and triage patients with OS to guide further treatment. To this end, we analyzed the clinical characteristics of patients with OS and also developed and validated a nomogram, aiming to provide a practical tool for rapid recognition of OSA in patients with COPD.

## Methods

### Study population

The patients confirmed with COPD presented in our emergency department from March 2017 to March 2022 due to a recent deterioration of cough, expectoration of phlegm, and shortness of breath were consecutively enrolled in this study. Exclusion criteria included those as follows: 1. Patients with other severe diseases which might also cause dyspnea, such as congestive heart failure, interstitial lung diseases, myasthenia gravis, and severe kidney or liver disease; 2. Patients with a history of NPPV dependency; 3. Patients with incomplete clinical data; 4. Pregnancy; and 5. patients who refuse to receive overnight sleep tests. A total of 330 participants were included, all of whom completed questionnaires and post-recovery overnight sleep cardiorespiratory monitoring. The subjects' medical history, laboratory chemistries, and other relevant information were recorded.

This study was approved by the Medical Ethics Committee of Tongji Medical College, Huazhong University of Science and Technology (2016S0130) and was conducted in accordance with the ethical standards outlined in the 1964 Declaration of Helsinki and subsequent amendments. All subjects signed a written informed consent form before participating in the study.

### Data collection

Demographic data including name, age, gender, body mass index (BMI), neck circumference (NC), and medical history were collected. Furthermore, blood samples and spirometry results were collected. The Global Initiative for Chronic Obstructive Lung Disease (GOLD) stage defined by the guideline was used to measure the severity of COPD (Singh et al., 2019).

### Questionnaires

All questionnaires used the validated version in Chinese. The modified Medical Research Council (mMRC) dyspnea scale was used to evaluate the degree of dyspnea, and an mMRC score of ≥2 was considered as the critical value of severity (Vogelmeier et al., 2017). The COPD assessment test (CAT) was used to assess the degree of health impairment. A CAT score of ≥10 prompts that medical intervention is needed (Kwon et al., 2013). The Sleep Apnea Clinical Score was used to evaluate the probability of OSA (Flemons et al., 1994), and a score of ≥5 suggests that sleep monitoring is recommended (Gali et al., 2009). The modified Epworth Sleepiness Scale was used to assess excessive daytime sleepiness (Johns, 1993; Zhang et al., 2011), and an mESS score of ≥10 is considered to be indicative of daytime sleepiness. Using the Pittsburgh Sleep Quality Index (PSQI) to estimate the quality of nighttime sleep (Buysse et al., 1989), with five points as the threshold, the lower the score is, the better the sleep quality is.

### Sleep study

A sleep study was not done until the patient's condition became stable when no more oxygen administration or NPPV was needed. The overnight cardiorespiratory monitoring was done by a portable monitoring (PM) device (type 3, Alice PDx, Respironics Inc. Murrysville, USA), and its accuracy has been experimentally confirmed (Nigro et al., 2013). The device includes a thermistor to monitor oronasal airflow and snoring, two bands for respiratory inductive plethysmograph determined by the ribcage and abdominal movements, a pulse oximeter, and an accelerometer to record body position. All sleep study records were manually scored by three experienced researchers (WW, SY, and PT) and validated by a senior expert (JZ) and conformed to the American Academy of Sleep Medicine (AASM) 2012 standards (Berry et al., 2012) and AASM position statement 2018 (Malhotra et al., 2018). The diagnosis of OSA can be established if the apnea-hypopnea index (AHI) is ≥5/h alone with typical clinical symptoms.

### Statistical analysis

SPSS statistical software (version 26.0, Chicago, IL, USA) and R software (version 4.2.1, http://www.Rproject.org) were used for analyses. Normally distributed continuous variables were represented by the mean ± standard deviation (SD), while non-normal continuous variables were expressed as the median (interquartile ranges). Categorical variables were reported as frequencies (percentages). Student's *t*-test, the Mann–Whitney U-test, the chi-square test, or Fisher's exact test were used where appropriate.

The “base” package of R was applied to randomly assign the patients to the training group and the validation group in the 7:3 ratio. In the training group, variables with a *p* < 0.05 in the univariate analysis were included in the multivariate logistic regression analysis, and the forward stepwise likelihood-ratio method was used to select the variables that were eventually included in the model. The method least absolute shrinkage and selection operator (LASSO) was performed by using the “glmnet” R package to eliminate highly correlated factors to ensure that the multivariable logistic regression model was not overfitting. In this study, the LASSO regression was only used to ensure that the multivariable logistic regression models were not overfitting rather than for variable selection and modeling. A nomogram of the risk-predictive model for OSA was developed from the regression purposeful variable by the “rms” package in R. Candidates in the validation group were used for assessing the discrimination and calibration of the nomogram. We conducted internal validation by bootstrapping using 1,000 replications to decrease the overfit bias, then the receiver operating characteristic (ROC) curve was constructed, and the area under the ROC curve (AUC) was employed to assess the model's discrimination. Calibration curves were plotted to assess the calibration of this model, accompanied by the Hosmer–Lemeshow test (*p* > 0.05 was considered as the goodness of calibration). Decision curve analysis (DCA) shows the standardized net benefit relative to the risk threshold probability and is used to evaluate the clinical utility of the model (Fitzgerald et al., 2015). The clinical impact curve analysis (CICA) shows the number of high-risk and true-positive patients at different threshold probabilities. A two-sided *p* < 0.05 was considered to be statistically significant.

## Results

### Characteristics of patients

We recruited 338 patients initially. A total of eight patients were excluded for the following reasons: those who refused overnight sleep tests (*n* = 3), those who have received NPPV therapies before (*n* = 1), those who are pregnant (*n* = 1), and those who have incomplete clinical data (*n* = 3). A total of 96 (29.1%) patients were diagnosed with OSA with a median age of 70 years. A total of 279 (84.5%) participants were men, 267 (80.9%) had a smoking history, and the common comorbidities among patients were hypertension (52.7%), type 2 diabetes (51.5%), coronary heart disease (20.0%), and hyperlipidemia (50.3%). As compared with patients with COPD alone, patients with COPD combined with OSA were overweight, had poorer sleep quality, less acute exacerbation (AE) of COPD in the prior year, more underlying diseases, but lower C-reactive protein (CRP) and better airway obstruction (all *p* < 0.05). A detailed comparison of clinical data between with and without OSA groups is shown in Table 1.

**Table 1:** Demographics and clinical characteristics of patients with COPD.

**Characteristics**	**All patients (*n* = 330)**	**COPD patients**		***p*-value**
		**Without OSA (*****n*** = **234)**	**With OSA (*****n*** = **96)**	
**Gender**				0.289
Male, *n* (%)	279 (84.5%)	201 (85.9%)	78 (81.2%)	
Female, *n* (%)	51 (15.5%)	33 (14.1%)	18 (18.8%)	
Age, years	70.0 (65.0, 77.0)	69.0 (65.0, 77.0)	71.5 (64.5, 77.5)	0.177
BMI, kg/m^2^	20.25 (17.70, 24.20)	18.90 (17.30, 22.70)	24.35 (21.55, 28.63)	< 0.001^*^
NC, cm	36 (34, 40)	34 (34, 38)	40 (38, 42)	< 0.001^*^
**Smoke**				0.305
Yes, *n* (%)	267 (80.9%)	186 (79.5%)	81 (84.4%)	
No, *n* (%)	63 (19.1%)	48 (20.5%)	15 (15.6%)	
**AE in the previous year**				< 0.001^*^
Yes, *n* (%)	240 (72.7%)	186 (79.5%)	54 (56.2%)	
No, *n* (%)	90 (27.3%)	48 (20.5%)	42 (43.8%)	
**Hypertension**				< 0.001^*^
Yes, *n* (%)	174 (52.7%)	105 (44.9%)	69 (71.9%)	
No, *n* (%)	156 (47.3%)	129 (55.1%)	27 (28.1%)	
**Type 2 diabetes**				< 0.001^*^
Yes, *n* (%)	170 (51.5%)	105 (44.9%)	65 (67.7%)	
No, *n* (%)	160 (48.5%)	129 (55.1%)	31 (32.3%)	
**Coronary heart disease**				< 0.001^*^
Yes, *n* (%)	66 (20.0%)	33 (14.1%)	33 (34.4%)	
No, *n* (%)	264 (80.0%)	201 (85.9%)	63 (65.6%)	
**Hyperlipidemia**				0.104
Yes, *n* (%)	166 (50.3%)	111 (47.4%)	55 (57.3%)	
No, *n* (%)	164 (49.7%)	123 (52.6%)	41 (42.7%)	
**Routine blood test**				
WBC, × 10^9^/L	7.06 (5.29, 10.30)	7.06 (5.29, 10.76)	7.04 (5.19, 8.64)	0.144
RBC, × 10^12^/L	4.39 (4.12, 4.81)	4.39 (4.07, 4.75)	4.40 (4.20, 4.93)	0.088
Hb, g/L	135.0 (122.8, 141.0)	135.0 (121.0, 141.0)	135.0 (123.0, 144.5)	0.833
HCT, %	41.60 (38.10, 44.70)	40.80 (37.80, 44.53)	42.20 (38.30, 45.30)	0.203
MCV, fl	92.00 (89.58, 97.30)	92.00 (89.48, 96.78)	92.50 (89.93, 99.25)	0.528
MCH, pg	30.20 (28.80, 31.90)	30.20 (28.80, 31.90)	29.80 (28.50, 31.58)	0.466
MCHC, g/L	323.0 (313.0, 332.0)	324.0 (313.0, 331.0)	319.0 (312.0, 333.5)	0.240
**Coagulation index**				
D-Dimer, ug/mL	0.79 (0.42, 2.03)	0.78 (0.42, 2.21)	0.79 (0.43, 1.43)	0.379
PT, s	13.7 (13.3, 14.7)	13.7 (13.1, 14.7)	13.9 (13.3, 15.3)	0.269
INR	1.08 (1.03, 1.26)	1.07 (1.01, 1.20)	1.13 (1.05, 1.28)	0.064
APTT, s	36.1 (32.9, 39.2)	36.0 (32.8, 38.8)	36.3 (33.5, 40.9)	0.023^*^
FIB, g/L	4.20 (3.19, 5.49)	4.26 (3.20, 5.80)	4.19 (3.17, 4.83)	0.025^*^
TT, s	18.20 (17.00, 19.73)	18.50 (17.20, 19.80)	17.75 (16.40, 19.10)	0.017^*^
**Blood gas analysis**				
PH	7.39 (7.32, 7.44)	7.39 (7.34, 7.44)	7.37 (7.29, 7.45)	0.073
PaO_2_, mmHg	78.0 (64.0, 90.0)	77.7 (63.5, 93.0)	78.0 (64.0, 86.0)	0.793
PaCO_2_, mmHg	46.70 (39.30, 66.40)	46.40 (39.00, 62.85)	50.40 (40.00, 71.80)	0.265
BNP, pg/mL	112.2 (57.0, 292.4)	128.1 (57.0, 350.5)	81.1 (57.0, 274.6)	0.317
CRP, mg/L	21.20 (4.79, 53.08)	24.40 (5.70, 73.61)	11.71(3.90, 33.12)	< 0.001^*^
**Questionnaires**				
mMRC, point	3 (2, 4)	3 (3, 4)	3 (2, 4)	< 0.001^*^
CAT, point	26 (21, 31)	26 (23, 31)	21 (15, 27)	< 0.001^*^
SACS, point	4.00 (3.00, 13.00)	3.00 (2.00, 6.00)	16.00 (8.25, 28.75)	< 0.001^*^
mESS, point	10.00 (7.00, 14.00)	10.00 (7.00, 13.00)	9.50 (5.00, 14.75)	0.532
PSQI, point	9.00 (6.00, 12.00)	9.00 (6.00, 12.00)	9.00 (6.25, 11.00)	0.722
**GOLD stage**, ***n*** **(%)**				< 0.001^*^
1	119 (36.1%)	82 (35.1%)	37 (38.5%)	
2	90 (27.3%)	60 (25.6%)	30 (31.3%)	
3	85 (25.7%)	62 (26.5%)	23 (23.9%)	
4	36(10.9%)	30 (12.8%)	6 (6.3%)	
**Sleep parameters**				
AHI, times per hr	6.60 (0.60, 18.30)	1.20 (0.60, 2.30)	18.10 (12.48, 33.15)	< 0.001^*^
ODI, times per hr	10.9 (1.3, 21.2)	1.6 (0.7, 2.9)	22.5 (18.2, 54.3)	< 0.001^*^
Mean SaO_2_, %	91.0 ± 3.7	92.7 ± 7.7	89.2 ± 4.6	< 0.001^*^
Minimum SaO_2_, %	75.2 ± 10.6	83.5 ± 6.9	72.03 ± 11.2	< 0.001^*^

COPD, chronic obstructive pulmonary disease; OSA, obstructive sleep apnea syndrome; BMI, body mass index; NC, neck circumference; AE: acute exacerbation; WBC, white blood cell; RBC, red blood cell; Hb, hemoglobin; HCT, hematocrit; MCV, mean corpuscular volume; MCH, mean corpuscular hemoglobin; MCHC, mean corpuscular hemoglobin concentration; PT, prothrombin time; INR, international normalized ratio; APTT, activated coagulation time of whole blood; FIB, fibrinogen; TT, thrombin time; PH, potential of hydrogen; PaO_2_, arterial oxygen partial pressure; PaCO_2_, arterial carbon dioxide pressure; BNP, brain natriuretic peptide; CRP, C-reactive protein; mMRC, modified Medical Research Council dyspnea scale; CAT, chronic obstructive pulmonary disease assessment test; SACS, sleep apnea clinical scale; mESS, modified Epworth sleepiness scale; PSQI, Pittsburgh Sleep Quality Index; GOLD, Global Initiative for Chronic Obstructive Lung Disease; AHI, apnea-hypopnea index; ODI, oxygen desaturation index; SaO_2_, arterial oxygen saturation. ^*^Means p < 0.05, the difference is statistically significant.

A total of 230 participants were randomly assigned to the training group and 100 to the validation group. Across the training and validation groups, 79.1 and 86.2% of patients with OSA, respectively, were men. In the training group, 67 (29.1%) patients were diagnosed with OSA, with a median age of 72 years. In the validation group, 29 (29.0%) patients were diagnosed with OSA, with a median age of 68 years. There were no significant differences in the features of demographic and clinical characteristics between training and validation groups (Supplementary Table 1). Table 2 summarizes the characteristics of patients with COPD with and without OSA of the training group and the validation group. Patients in both OSA groups revealed a higher proportion of hypertension, type 2 diabetes, and coronary heart disease; higher BMI, NC, SACS; lower CRP, mMRC, and CAT; as well as poorer polysomnographic data and less AE (all *p* < 0.05). The differences in airflow limitation between OS and COPD groups in training and validation groups were statistically significant (*p* < 0.05). Participants who experienced more AE showed worse airflow limitation and poorer health status.

**Table 2:** Demographics and clinical characteristics of the training group and the validation group.

**Characteristics**	**Training group (*****n*** = **230)**			**Validation group (*****n*** = **100)**		
	**without OSA (*****n*** = **163)**	**with OSA (*****n*** = **67)**	***p*** **-value**	**without OSA (*****n*** = **71)**	**with OSA (*****n*** = **29)**	***p-*** **value**
**Gender**			0.203			0.970
Male, *n* (%)	140 (85.9%)	53 (79.1%)		61 (85.9%)	25 (86.2%)	
Female, *n* (%)	23 (14.1%)	14 (20.9%)		10 (14.1%)	4 (13.8%)	
Age, years	68 (64, 76)	72 (68, 78)	0.013^*^	71 (65, 79)	68.0 (57.5, 74.0)	0.200
BMI, kg/m^2^	18.9 (17.3, 22.1)	24.3 (21.7, 28.7)	< 0.001^*^	18.8 (17.6, 23.0)	24.4 (20.2, 26.8)	< 0.001^*^
NC, cm	34 (34, 38)	42 (38, 44)	< 0.001^*^	36 (34, 38)	38 (38,42)	< 0.001^*^
**Smoke**			0.503			0.396
Yes, *n* (%)	130 (80.0%)	56 (83.6%)		56 (78.9%)	25 (86.2%)	
No, *n* (%)	33(20.0%)	11 (16.4%)		15 (21.1%)	4 (13.8%)	
**AE in the previous year**			0.007^*^			< 0.001^*^
Yes, *n* (%)	128 (78.5%)	41 (61.2%)		58 (81.7%)	13 (44.8%)	
No, *n* (%)	35 (21.5%)	26 (38.8%)		13 (18.3%)	16 (55.2%)	
**Hypertension**			< 0.001^*^			0.028^*^
Yes, *n* (%)	78 (47.9%)	51 (76.1%)		27 (38.0%)	18 (62.1%)	
No, *n* (%)	85 (52.1%)	16 (23.9%)		44 (62.0%)	11 (37.9%)	
**Type 2 diabetes**			0.002^*^			0.035^*^
Yes, *n* (%)	75 (46.0%)	46 (68.7%)		30 (42.3%)	19 (65.5%)	
No, *n* (%)	88 (56.0%)	21 (31.3%)		41 (57.7%)	10 (34.5%)	
**Coronary heart disease**			0.001^*^			0.016^*^
Yes, *n* (%)	20 (12.3%)	21 (31.3%)		13 (18.3%)	12 (41.4%)	
No, *n* (%)	143 (87.7%)	46 (68.7%)		58 (81.7%)	17 (58.6%)	
**Hyperlipidemia**			0.175			0.364
Yes, *n* (%)	74 (45.4%)	37 (55.2%)		37 (52.1%)	18 (62.1%)	
No, *n* (%)	89 (54.6%)	30(44.8%)		34 (47.9%)	11 (37.9%)	
**Routine blood test**						
WBC, × 10^9^/L	7.06 (5.28, 10.69)	6.92 (5.19, 8.64)	0.273	7.79 (5.38, 11.49)	7.58 (5.39, 8.87)	0.335
RBC, × 10^12^/L	4.40 (4.07, 4.74)	4.39 (4.20, 4.93)	0.251	4.35 (4.03, 4.80)	4.58 (4.26, 5.16)	0.194
Hb, g/L	135 (123, 141)	134 (123, 143)	0.586	135 (119, 141)	136 (123, 152)	0.236
HCT, %	40.8 (38.2, 44.5)	42.2 (38.2, 45.0)	0.592	40.4 (36.4, 44.6)	41.9 (38.4, 46.5)	0.132
MCV, fl	92.0 (89.6, 97.0)	92.5 (89.9, 100.6)	0.698	91.7 (88.2, 96.7)	92.5 (90.4, 96.9)	0.533
MCH, pg	30.2 (28.8, 31.9)	29.6 (28.5, 31.1)	0.189	30.2 (28.8, 32.2)	30.9 (28.4, 32.0)	0.556
MCHC, g/L	325.0 (314.0, 333.0)	318.0 (312.0, 332.0)	0.076	324.0 (313.0, 330.0)	322.0 (312.5, 339.5)	0.659
**Coagulation index**						
D-Dimer, ug/mL	1.06 (0.42, 2.39)	0.81 (0.47, 1.61)	0.716	0.57 (0.46, 1.95)	0.75 (0.36, 1.33)	0.334
PT, s	13.7 (13.3, 14.7)	13.9 (13.3, 15.6)	0.182	13.7 (12.8, 15.0)	13.7 (13.2, 14.5)	0.897
INR	1.07 (1.03, 1.20)	1.09 (1.05, 1.28)	0.193	1.09 (0.99, 1.28)	1.15 (1.07, 1.27)	0.197
APTT, s	36.0 (32.8, 39.1)	36.3 (34.7, 39.6)	0.087	35.1 (33.0, 37.9)	37.4 (32.9, 41.7)	0.141
FIB, g/L	4.33 (3.23, 5.80)	4.19 (3.17, 4.78)	0.029^*^	4.03 (3.10, 6.07)	4.20 (3.11, 5.00)	0.466
TT, s	18.4 (17.1, 19.8)	17.6 (16.4, 19.4)	0.038^*^	18.6 (17.3, 19.8)	17.8 (17.3, 18.9)	0.189
**Blood gas analysis**						
PH	7.39 (7.34, 7.44)	7.37 (7.29, 7.45)	0.178	7.39 (7.35, 7.44)	7.37 (7.30, 7.45)	0.200
PaO_2_, mmHg	77.4 (62.0, 92.0)	78.0 (64.0, 87.0)	0.797	80.0 (64.0, 95.0)	78.0 (63.5, 86.0)	0.362
PaCO_2_, mmHg	46.4 (38.1, 65.7)	52.7 (40.0, 71.8)	0.338	45.4 (39.3, 61.9)	46.7 (39.1, 70.8)	0.608
BNP, pg/mL	120.5 (57.0, 292.4)	107.4 (55.1, 292.4)	0.803	135.9 (57.0, 352.4)	64.5 (57.8, 173.4)	0.177
CRP, mg/L	24.40 (6.27, 73.61)	10.98 (3.90, 23.88)	< 0.001^*^	23.88 (2.52, 89.40)	12.43 (4.34, 34.70)	< 0.001^*^
**Questionnaires**						
mMRC, point	3 (3, 4)	3 (2, 4)	0.007^*^	3 (3, 4)	3 (2, 3)	0.005^*^
CAT, point	26 (23, 32)	22 (17, 27)	< 0.001^*^	26 (23, 31)	20 (15, 28)	0.001^*^
SACS, point	3 (2, 6)	18 (9, 30)	< 0.001^*^	3 (2, 6)	16 (7, 18)	< 0.001^*^
mESS, point	11 (8, 14)	10 (5, 15)	0.607	10 (6, 13)	9 (5, 15)	0.766
PSQI, point	8 (6, 12)	9 (7, 11)	0.521	9 (6, 12)	9 (6, 11)	0.771
**GOLD Stage**, ***n*** **(%)**			< 0.001^*^			< 0.001^*^
1	57 (35.0%)	25 (37.3%)		26 (36.6%)	7 (24.1%)	
2	45 (27.6%)	20 (29.8%)		13 (18.3%)	11 (37.9%)	
3	43 (26.4%)	17 (25.4%)		19 (26.8%)	7 (24.1%)	
4	18 (11.0%)	5 (7.5%)		13 (18.3%)	4 (13.9%)	
**Sleep parameters**						
AHI (times per hr)	1.1 (0.3, 2.5)	12.6 (6.3, 17.8)	< 0.001^*^	1.2 (0.4, 2.7)	14.2 (8.1, 19.8)	< 0.001^*^
ODI (times per hr)	1.6 (0.6, 3.5)	41.2 (18.13, 63.5)	< 0.001^*^	1.8 (0.7, 3.5)	38.2 (18.2, 63.0)	< 0.001^*^
Mean SaO_2_ (%)	95.6 ± 2.2	93.7 ± 3.4	< 0.001^*^	94.9 ± 4.2	91.4 ± 3.0	< 0.001^*^
Minimum SaO_2_ (%)	87.50 ± 7.70	71.33 ± 11.32	< 0.001^*^	87.60 ± 4.81	73.66± 10.89	< 0.001^*^

COPD, chronic obstructive pulmonary disease; OSA, obstructive sleep apnea syndrome; BMI, body mass index; NC, neck circumference; AE: acute exacerbation; WBC, white blood cell; RBC, red blood cell; Hb, hemoglobin; HCT, hematocrit; MCV, mean corpuscular volume; MCH, mean corpuscular hemoglobin; MCHC, mean corpuscular hemoglobin concentration; PT, prothrombin time; INR, international normalized ratio; APTT, activated coagulation time of whole blood; FIB, fibrinogen; TT, thrombin time; PH, potential of hydrogen; PaO2, arterial oxygen partial pressure; PaCO2, arterial carbon dioxide pressure; BNP, brain natriuretic peptide; CRP, C-reactive protein; mMRC, modified Medical Research Council dyspnea scale; CAT, chronic obstructive pulmonary disease assessment test; SACS, sleep apnea clinical scale; mESS, modified Epworth sleepiness scale; PSQI, Pittsburgh Sleep Quality Index; GOLD, Global Initiative for Chronic Obstructive Lung Disease; AHI, apnea-hypopnea index; ODI, oxygen desaturation index; SaO_2_, arterial oxygen saturation.

### Construction of the nomogram

**Figure 1:**
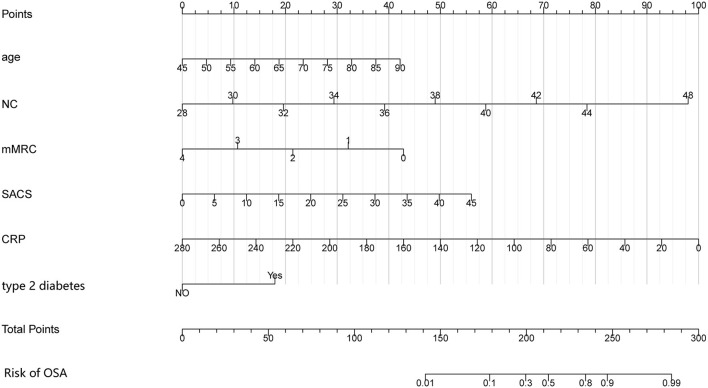
Nomogram for predicting the risk of OSA in patients with COPD. The nomogram included six risk factors: age, NC, type 2 diabetes, mMRC questionnaire, SACS questionnaire, and CRP. The patient's status for each predictor is plotted on a horizontal scale, and vertical lines are drawn to obtain the corresponding points. After all points have been summed, the total score on the total point line is plotted and a vertical line is drawn down to the accompanying line labeled Risk of OSA. The point where this line crosses the accompanying line indicates the predicted probability of diagnosis. COPD, chronic obstructive pulmonary disease; OSA, obstructive sleep apnea syndrome; NC, neck circumference; mMRC, modified Medical Research Council; SACS, Sleep Apnea Clinical Score; CRP, C-reactive protein.

**Figure 2:**
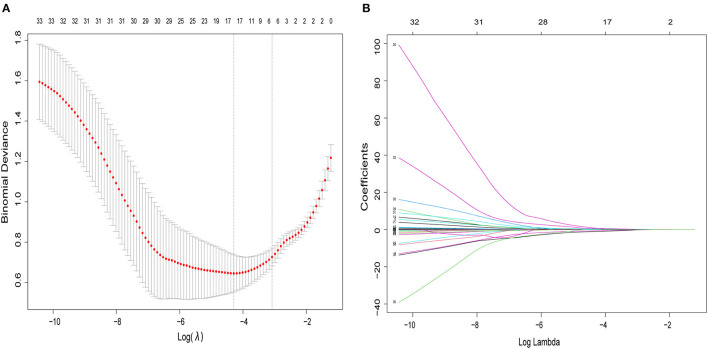
The variables filtering process of the LASSO regression. In order to avoid overfitting, the LASSO regression suggested including six variables when merging OSA was the endpoint. In the variable selection process, first of all, the univariate analysis was used to select potential factors. Then, based on these potential factors, the multivariable logistic regression model was constructed. In this study, the LASSO regression was only used to ensure that the multivariable logistic regression models were not overfitting rather than for variable selection and modeling. **(A)** Optimal parameter (lambda) selection in the LASSO logistic regression used 10-fold cross-validation *via* minimum criteria. The dotted vertical lines were drawn at the best values using the minimum criteria and 1 standard error of the minimum criteria (the 1-SE criteria). **(B)** LASSO coefficient profiles of the 34 features. A coefficient profile plot was produced against the log (lambda) sequence. LASSO, least absolute shrinkage and selection operator; SE, standard error.

The multivariate logistic regression model considered 14 parameters with a *p* < 0.05 in the univariate analysis, including age, BMI, NC, hypertension, type 2 diabetes, coronary heart disease, AE in the previous year, fibrinogen, thrombin time, CRP, mMRC, CAT, SACS, and GOLD stage. The multivariate logistic analysis showed six independent risk-predictive factors for OS to develop nomogram: age (OR: 1.062, 1.003–1.124), type 2 diabetes (OR: 3.166, 1.263–7.939), NC (OR: 1.370, 1.098–1,709), mMRC (OR: 0.503, 0.325–0.777), SACS (OR: 1.083, 1.004–1.168), and CRP (OR: 0.977, 0.962–0.993) (Table 3, Figure 1). All variables eventually incorporated into the multivariate model were essential to the modeling process in the LASSO regression (Figure 2). LASSO regression minimized the influence of multicollinearity and had the advantages of strong predictability and high robustness. We identified independent factors in the training group by non-zero coefficients in the LASSO regression, and optimal parameter (lambda) selection in the LASSO model used 10-fold cross-validation *via* minimum criteria.

**Table 3:** Multivariate logistic regression analysis in train group.

**Variable**	**β coefficient**	**Standard error**	**Wald**	**OR (95%CI)**	***p-*value**
Age	0.060	0.029	4.314	1.062 (1.003–1.124)	0.038
Type 2 diabetes	1.153	0.469	6.038	3.166 (1.263–7.939)	0.014
NC	0.315	0.113	7.761	1.370 (1.098–1.709)	0.005
mMRC	−0.688	0.222	9.578	0.503 (0.325–0.777)	0.002
SACS	0.080	0.039	4.299	1.083 (1.004–1.168)	0.038
CRP	−0.023	0.008	7.861	0.977 (0.962–0.993)	0.005
Constants	−16.016	4.488	12.735	0.000	< 0.001

CI, confidence interval; OR, odds ratio; NC, neck circumference; mMRC, modified Medical Research Council dyspnea scale; SACS, sleep apnea clinical scale; CRP, C-reactive protein. ^*^Means p < 0.05, the difference is statistically significant.

### Validation of the nomogram

**Figure 3:**
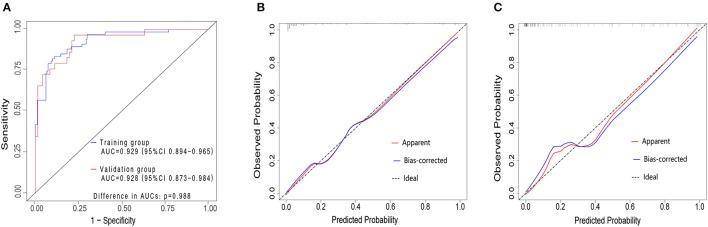
ROC curves in both the training and validation cohorts **(A)**. Training group (blue curve), AUC = 0.929 (95%CI 0.894–0.965). Validation group (red curve), AUC = 0.928 (95%CI 0.873–0.984) The AUCs for the OSA in COPD consecutive patients in the training and validation group exceed 0.7, which demonstrated that the nomogram could accurately predict the risk of OSA in consecutive patients with COPD. Calibration plot in the training group **(B)** and validation group **(C)**. The y-axis represents the actual probability of patients with OSA as validated by the sleep monitoring study, and the x-axis represents the predicted risk of OSA by the risk nomogram. The dotted line represents a perfect prediction by an ideal model, while the blue line represents the performance of the risk nomogram. A closer fit of the blue line to the dotted line represents a better prediction. COPD, chronic obstructive pulmonary disease; OSA, obstructive sleep apnea syndrome; ROC, receiver operating characteristic curves; AUC, area under the ROC curve; CI, confidence interval.

The validation of the nomogram was performed with a 1,000 bootstrap analysis. The nomogram yielded relatively high AUCs in both the training group [0.929, 95% confidence interval (CI) 0.894–0.965] and validation group (0.928, 95%CI 0.873–0.984), exceeding 0.7 in both cases, indicating a satisfactory performance (Figure 3A). Moreover, observations and predictions of OS correlated well with the calibration plots (Figures 3B, C). The Hosmer–Lemeshow test also showed that there was no significant statistical difference in both the training group (χ^2^ = 13.552, *p* = 0.139) and the validation group (χ^2^ = 10.710, *p* = 0.296), suggesting that the nomogram was well-calibrated.

### Clinical application

**Figure 4:**
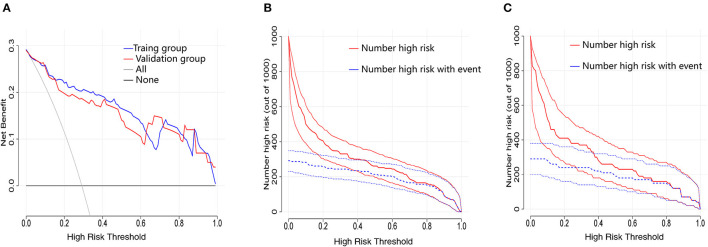
Evaluation of the clinical utility of nomogram prediction models in the training and validation groups. **(A)** Decision curves in both the training and validation groups. **(B)** Clinical impact curve in the training group. **(C)** Clinical impact curve in the validation group.

Decision curve analysis is a method to assess the benefits of a diagnostic test by quantifying the net benefit at different threshold probabilities to determine the clinical usefulness of the nomogram. Compared to the two thresholds of “no intervention” and “intervention for all,” both the training and validation groups displayed higher clinical net benefit (Figure 4A). The clinical impact curves for the training group (Figure 4B) and the validation group (Figure 4C) also showed good predictability and clinical utility.

## Discussion

Currently, OSA in the context of COPD is common with little attention. The prevalence of COPD combined with OSA varies from 2.9 to 65.9% (Shawon et al., 2017), and there is increasing evidence that patients with COPD are more likely to suffer from OSA than the general population of the same age (Sacks et al., 2018; Zhang et al., 2022). COPD patients with OSA tend to have more basic diseases and higher mortality than COPD-only patients, but the costly and time-consuming PSG and the possible bias of questionnaires may lead to underdiagnosis. Therefore, it is important to predict and diagnose OSA early. This study revealed the incidence of OSA as well as the risk factors for developing OSA. These terms are readily available and have good predictive performance, which is suitable for use in outpatients and hospitals without PSG. Our nomogram shows good discrimination and sufficient prediction performance, therefore, proving it to be robust.

We observed that of the 330 patients with COPD, the prevalence of OSA was 29.1%, which was at a relatively moderate level. Compared with patients with COPD alone, patients combined with OSA were overweight, had lower CRP, better airway obstruction, and less AE during the 12 months before enrolling into the study, and were more likely to have type 2 diabetes. It has been reported in the literature that OSA is very common in patients with type 2 diabetes, 55–86% of whom have OSA (Schipper et al., 2021). Obstructive sleep apnea syndrome, through the effects of intermittent hypoxemia and sleep fragmentation, could contribute to the development of type 2 diabetes (Aurora and Punjabi, 2013). Meanwhile, age and obesity are well-known predictors of OSA. Studies revealed that the incidence of OSA was positively correlated with age (Fietze et al., 2019; Lyons et al., 2020). Older adults might have reduced tethering of the upper airway by lung volume because of loss of elastic recoil in the lung or have a more easily collapsible airway caused by the loss of collagen. Moreover, the efficiency of the upper airway dilator muscles might fall with age (Eikermann et al., 2007; Liu et al., 2021). Body mass index, as an indicator of obesity, reflects overall fat distribution but does not adequately take into account neck fat distribution, which has limitations. Obesity could increase the likelihood of airway collapse by directly affecting the anatomy of the upper airway as fat is deposited in the neck (Schwartz et al., 2010; McNicholas and Pevernagie, 2022). Some studies showed that neck fat is thicker in OSA than in non-apnea snorers (Morinigo et al., 2022). Therefore, compared with traditional obesity evaluation, such as BMI, NC is a more accurate independent predictor of OSA (Simpson et al., 2010; Cho et al., 2016; Gasa et al., 2019).

In patients with COPD, an increase in breathing as a result of small (< 2 mm) airway obstruction, muscle contraction, and elastic recoil of the lung instigate symptoms of dyspnea (Rabe, 2006; O'Donnell et al., 2007). A review proposed the “obesity paradox” and speculated its possible mechanism, which concluded that obese patients with COPD have better dyspnea scores than non-obese patients (Guenette et al., 2010). Furthermore, the prevalence of OSA has gradually increased with the epidemic of obesity according to epidemiological data (Young et al., 1993; Peppard et al., 2013); that is, non-obese patients with COPD have more severe dyspnea but a lower probability of combined OSA than obese patients. This is consistent with the results from our study where patients with COPD alone have higher mMRC scores. This parameter can be used as an independent factor and diagnostic criterium of OSA.

C-reactive protein has been proven to be an effective inflammatory biomarker during COPD exacerbation. It was reported that CRP concentrations were found to be consistently elevated in the AE state and were significantly higher than in healthy or stable controls (Valipour et al., 2008; Lin et al., 2019). Meanwhile, a high level of CRP was related to the risk of AE (Cano et al., 2004; Thomsen et al., 2013). It is valuable in the confirmation of COPD exacerbation when combined with a major exacerbation symptom (Hurst et al., 2006). This is consistent with our results that CRP was inversely correlated with the incidence of OSA. Notably, patients with COPD alone had more AE in the prior year in our research though some reports held opposite opinions (Marin et al., 2010; Donovan et al., 2019; Hong et al., 2020). The reason for this phenomenon is possibly that patients with COPD in our study were with an AE state and had lower BMI (median:18.90), compared with patients with COPD from Western countries (the median BMI of most patients with COPD is ≥25 kg/m^2^). The loss of body weight is a common problem in patients with COPD (Engelen et al., 1994, 1999). BMI was correlated with pulmonary function positively and exacerbations negatively (Cano et al., 2004; Wu et al., 2018). However, studies should be more and deeper to verify our results.

Among the available screening tools for detecting OSA, although these questionnaires were validated in the general population, they were found to have limited sensitivity and specificity in specific populations. Xiong et al., in a 2019 study on five questionnaires in screening COPD patients with OSA showed that SACS had a moderate predictive value in screening severe OSA, with an AUC of 0.750 (Xiong et al., 2019). While Wang et al. (2021) showed that SACS had excellent sensitivity (93.4–94.6%) and a negative predictive value (77.3–90.9%) in evaluating the prevalence of OSA in patients with COPD. In our study, SACS has a good predictive value, but there are few studies on the predictive value of SACS in COPD patients with OSA, and more studies are still needed.

We construct a nomogram in which all predictors are common demographic and anthropometry measures and questionnaires that could be obtained in outpatient without additional testing, greatly reducing the burden on physicians and patients, and facilitating the clinical procedure for OS diagnosis. There are some limitations to this study. First, this is a single-center study, where training and validation groups are recruited from the same center. Multicenter studies should be developed to validate our results. Second, our samples are relatively small. Third, we used the PM device. However, previous studies (Parra et al., 1997; Vat et al., 2015) showed good consistency and correlation between the PM device and PSG results.

In conclusion, we developed and validated a new nomogram model, which consisted of six independent risk factors for OSA, which may empower clinicians and patients with COPD with earlier, more accurate information regarding the risk of OSA.

## Data availability statement

The original contributions presented in the study are included in the article/Supplementary material, further inquiries can be directed to the corresponding authors.

## Ethics statement

The studies involving human participants were reviewed and approved by Medical Ethics Committee of Tongji Medical College, Huazhong University of Science and Technology. The patients/participants provided their written informed consent to participate in this study.

## Author contributions

TP, WW, SY, and JZ conceived and designed the study. TP, WW, and SY collected the data. TP, ZL, YY, ZH, and RN participated in the investigation of the study. TP analyzed the data. TP and JZ were responsible for data interpretation. TP, WW, AJ, XW, and JZ wrote the initial draft of the manuscript. SY, ZL, YY, ZH, and RN involved in revising the manuscript. All authors contributed to the study and also read and approved the final manuscript.

## References

[B1] Aurora R. N. Punjabi N. M. (2013). Obstructive sleep apnoea and type 2 diabetes mellitus: a bidirectional association. Lancet Resp. Med. 1, 329–338. 10.1016/S2213-2600(13)70039-0 24429158

[B2] Berry R. B. Budhiraja R. Gottlieb D. J. Gozal D. Iber C. Kapur V. K.. (2012). Rules for scoring respiratory events in sleep: update of the 2007 AASM manual for the scoring of sleep and associated events. Deliberations of the sleep apnea definitions task force of the American academy of sleep medicine. J. Clin. Sleep Med. 8, 597–619. 10.5664/jcsm.2172 23066376 PMC3459210

[B3] Buist A. S. McBurnie M. A. Vollmer W. M. Gillespie S. Burney P. Mannino D. M.. (2007). International variation in the prevalence of COPD (the BOLD Study): a population-based prevalence study. Lancet 370, 741–750. 10.1016/S0140-6736(07)61377-4 17765523

[B4] Buysse D. J. Reynolds C. F. Monk T. H. Berman S. R. Kupfer D. J. (1989). The pittsburgh sleep quality index: a new instrument for psychiatric practice and research. Psychiatry Res. 28, 193–213. 10.1016/0165-1781(89)90047-4 2748771

[B5] Cano N. J. M. Pichard C. Roth H. Court-Fortuné I. Cynober L. Gérard-Boncompain M.. (2004). C-reactive protein and body mass index predict outcome in end-stage respiratory failure. Chest 126, 540–546. 10.1378/chest.126.2.540 15302742

[B6] Chiu H. Y. Chen P.-Y. Chuang L.-P. Chen N.-H. Tu Y.-K. Hsieh Y.-J.. (2017). Diagnostic accuracy of the Berlin questionnaire, STOP-BANG, STOP, and Epworth sleepiness scale in detecting obstructive sleep apnea: a bivariate meta-analysis. Sleep Med. Rev. 36, 57–70. 10.1016/j.smrv.2016.10.004 27919588

[B7] Cho J. H. Choi J. H. Suh J. D. Ryu S. Cho S. H. (2016). Comparison of anthropometric data between asian and caucasian patients with obstructive sleep apnea: a meta-analysis. Clin. Exp. Otorhinolaryngol. 9, 1–7. 10.21053/ceo.2016.9.1.1 26976019 PMC4792237

[B8] Donovan L. M. Feemster L. C. Udris E. M. Griffith M. F. Spece L. J. Palen B. N.. (2019). Poor outcomes among patients with chronic obstructive pulmonary disease with higher risk for undiagnosed obstructive sleep apnea in the LOTT Cohort. J. Clin. Sleep Med. 15, 71–77. 10.5664/jcsm.7574 30621828 PMC6329538

[B9] Eikermann M. Jordan A. S. Chamberlin N. L. Gautam S. Wellman A. Lo Y.-L.. (2007). The influence of aging on pharyngeal collapsibility during sleep. Chest 131, 1702–1709. 10.1378/chest.06-2653 17413053 PMC2278166

[B10] Engelen M. P. Schols A. M. Baken W. C. Wesseling G. J. Wouters E. F. (1994). Nutritional depletion in relation to respiratory and peripheral skeletal muscle function in out-patients with COPD. Eur. Respir. J. 7, 1793–1797. 10.1183/09031936.94.07101793 7828687

[B11] Engelen M. P. Schols A. M. Lamers R. J. Wouters E. F. (1999). Different patterns of chronic tissue wasting among patients with chronic obstructive pulmonary disease. Clin. Nutr. 18, 275–280. 10.1016/S0261-5614(98)80024-1 10601534

[B12] Fietze I. Laharnar N. Obst A. Ewert R. Felix S. B. Garcia C.. (2019). Prevalence and association analysis of obstructive sleep apnea with gender and age differences - results of SHIP-Trend. J. Sleep Res. 28, e12770. 10.1111/jsr.12770 30272383

[B13] Fitzgerald M. Saville B. R. Lewis R. J. (2015). Decision curve analysis. JAMA 313, 409–410. 10.1001/jama.2015.37 25626037

[B14] Flemons W. W. Whitelaw W. A. Brant R. Remmers J. E. (1994). Likelihood ratios for a sleep apnea clinical prediction rule. Am. J. Resp. Crit. Care Med. 150, 1279–1285. 10.1164/ajrccm.150.5.7952553 7952553

[B15] Gali B. Whalen F. X. Schroeder D. R. Gay P. C. Plevak D. J. (2009). Identification of patients at risk for postoperative respiratory complications using a preoperative obstructive sleep apnea screening tool and postanesthesia care assessment. Anesthesiology 110, 869–877. 10.1097/ALN.0b013e31819b5d70 19293694

[B16] Gasa M. López-Padrós C. Monasterio C. Salord N. Mayos M. Vilarrasa N.. (2019). Anthropometrical phenotypes are important when explaining obstructive sleep apnea in female bariatric cohorts. J. Sleep Res. 28, e12830. 10.1111/jsr.12830 30740836

[B17] Guenette J. A. Jensen D. O'Donnell D. E. (2010). Respiratory function and the obesity paradox. Curr. Opin. Clin. Nutr. Metab. Care 13, 618–624. 10.1097/MCO.0b013e32833e3453 20975350

[B18] Hong Y. D. Onukwugha E. Slejko J. F. (2020). The economic burden of comorbid obstructive sleep apnea among patients with chronic obstructive pulmonary disease. J. Manag. Care Special. Pharmacy 26, 1353–1362. 10.18553/jmcp.2020.26.10.1353 32996389 PMC10391088

[B19] Hurst J. R. Donaldson G. C. Perera W. R. Wilkinson T. M. A. Bilello J. A. Hagan G. W.. (2006). Use of plasma biomarkers at exacerbation of chronic obstructive pulmonary disease. Am. J. Respir. Crit. Care Med. 174, 867–874. 10.1164/rccm.200604-506OC 16799074

[B20] Johns M. W. (1993). Daytime sleepiness, snoring, and obstructive sleep apnea. Epworth Sleep. Scale. Chest 103, 30–36. 10.1378/chest.103.1.30 8417909

[B21] Kwon N. Amin M. Hui D. S. Jung K.-S. Lim S. Y. Ta H. D.. (2013). Validity of the COPD assessment test translated into local languages for Asian patients. Chest 143, 703–710. 10.1378/chest.12-0535 23460156

[B22] Lin T. L. Chen W.-W. Ding Z.-R. Wei S.-C. Huang M.-L. Li C.-H. (2019). Correlations between serum amyloid A, C-reactive protein and clinical indices of patients with acutely exacerbated chronic obstructive pulmonary disease. J. Clin. Lab. Anal. 33, e22831. 10.1002/jcla.22831 30666727 PMC6528583

[B23] Liu Y. Zou J. Qian Y. Xu H. Zhu H. Meng L.. (2021). The association between obesity indices and obstructive sleep apnea is modified by age in a sex-specific manner. Sleep Breath. 25, 189–197. 10.1007/s11325-020-02083-4 32367469

[B24] Lyons M. M. Bhatt N. Y. Pack A. I. Magalang U. J. (2020). Global burden of sleep-disordered breathing and its implications. Respirology 25, 690–702. 10.1111/resp.13838 32436658

[B25] Malhotra R. K. Kirsch D. B. Kristo D. A. Olson E. J. Aurora R. N. Carden K. A.. (2018). Polysomnography for obstructive sleep apnea should include arousal-based scoring: an American academy of sleep medicine position statement. J. Clin. Sleep Med. 14, 1245–1247. 10.5664/jcsm.7234 29991439 PMC6040795

[B26] Marin J. M. Soriano J. B. Carrizo S. J. Boldova A. Celli B. R. (2010). Outcomes in patients with chronic obstructive pulmonary disease and obstructive sleep apnea: the overlap syndrome. Am. J. Respir. Crit. Care Med. 182, 325–331. 10.1164/rccm.200912-1869OC 20378728

[B27] McNicholas W. T. Pevernagie D. (2022). Obstructive sleep apnea: transition from pathophysiology to an integrative disease model. J. Sleep Res. 31, e13616. 10.1111/jsr.13616 35609941 PMC9539471

[B28] Morinigo R. Quraishi S. A. Ewing S. Azocar R. J. Schumann R. (2022). The B-APNEIC score: distilling the STOP-Bang questionnaire to identify patients at high risk for severe obstructive sleep apnoea. Anaesthesia 77, 286–292. 10.1111/anae.15571 34473837

[B29] Nigro C. A. Dibur E. Malnis S. Grandval S. Nogueira F. (2013). Validation of ApneaLink Ox™ for the diagnosis of obstructive sleep apnea. Sleep Breath. 17, 259–266. 10.1007/s11325-012-0684-4 22447171

[B30] O'Donnell D. E. Banzett R. B. Carrieri-Kohlman V. Casaburi R. Davenport P. W. Gandevia S. C.. (2007). Pathophysiology of dyspnea in chronic obstructive pulmonary disease: a roundtable. Proc. Am. Thorac. Soc. 4, 145–168. 10.1513/pats.200611-159CC 17494725

[B31] Parra O. García-Esclasans N. Montserrat J. M. García Eroles L. Ruíz J. López J. A.. (1997). Should patients with sleep apnoea/hypopnoea syndrome be diagnosed and managed on the basis of home sleep studies? Eur. Respir. J. 10, 1720–1724. 10.1183/09031936.97.10081720 9272909

[B32] Peppard P. E. Young T. Barnet J. H. Palta M. Hagen E. W. Hla K. M. (2013). Increased prevalence of sleep-disordered breathing in adults. Am. J. Epidemiol. 177, 1006–1014. 10.1093/aje/kws342 23589584 PMC3639722

[B33] Rabe K. F. (2006). Improving dyspnea in chronic obstructive pulmonary disease: optimal treatment strategies. Proc. Am. Thorac. Soc. 3, 270–275. 10.1513/pats.200601-002SF 16636097

[B34] Sacks D. Baxter B. Campbell B. C. V. Carpenter J. S. Cognard C. Dippel D.. (2018). Multisociety consensus quality improvement revised consensus statement for endovascular therapy of acute ischemic stroke. Int. J. Stroke 13, 612–632. 10.1177/1747493018778713 29786478

[B35] Schipper S. B. J. Van Veen M. M. Elders P. J. M. van Straten A. Van Der Werf Y. D. Knutson K. L.. (2021). Sleep disorders in people with type 2 diabetes and associated health outcomes: a review of the literature. Diabetologia 64, 2367–2377. 10.1007/s00125-021-05541-0 34401953 PMC8494668

[B36] Schwartz A. R. Patil S. P. Squier S. Schneider H. Kirkness J. P. Smith P. L. (2010). Obesity and upper airway control during sleep. J. Appl. Physiol. 108, 430–435. 10.1152/japplphysiol.00919.2009 19875707 PMC2822668

[B37] Shawon M. S. R. Perret J. L. Senaratna C. V. Lodge C. Hamilton G. S. Dharmage S. C. (2017). Current evidence on prevalence and clinical outcomes of co-morbid obstructive sleep apnea and chronic obstructive pulmonary disease: a systematic review. Sleep Med. Rev. 32, 58–68. 10.1016/j.smrv.2016.02.007 28169105

[B38] Simpson L. Mukherjee S. Cooper M. N. Ward K. L. Lee J. D. Fedson A. C.. (2010). Sex differences in the association of regional fat distribution with the severity of obstructive sleep apnea. Sleep 33, 467–474. 10.1093/sleep/33.4.467 20394315 PMC2849785

[B39] Singh D. Agusti A. Anzueto A. Barnes P. J. Bourbeau J. Celli B. R.. (2019). Global strategy for the diagnosis, management, and prevention of chronic obstructive lung disease: the GOLD science committee report 2019. Eur. Respir. J. 53, 1900164. 10.1183/13993003.00164-2019 30846476

[B40] Sterling K. L. Pépin J.-L. Linde-Zwirble W. Chen J. Benjafield A. V. Cistulli P. A.. (2022). Impact of positive airway pressure therapy adherence on outcomes in patients with obstructive sleep apnea and chronic obstructive pulmonary disease. Am. J. Respir. Crit. Care Med. 206, 197–205. 10.1164/rccm.202109-2035OC 35436176 PMC9887426

[B41] Suri T. M. Suri J. C. (2021). A review of therapies for the overlap syndrome of obstructive sleep apnea and chronic obstructive pulmonary disease. FASEB BioAdv. 3, 683–693. 10.1096/fba.2021-00024 34485837 PMC8409567

[B42] Tang M. Wang Y. Wang M. Tong R. Shi T. (2021). Risk for cardiovascular disease and one-year mortality in patients with chronic obstructive pulmonary disease and obstructive sleep apnea syndrome overlap syndrome. Front. Pharmacol. 12, 767982. 10.3389/fphar.2021.767982 34764876 PMC8576345

[B43] Thomsen M. Ingebrigtsen T. S. Marott J. L. Dahl M. Lange P. Vestbo J.. (2013). Inflammatory biomarkers and exacerbations in chronic obstructive pulmonary disease. JAMA 309, 2353–2361. 10.1001/jama.2013.5732 23757083

[B44] Valipour A. Schreder M. Wolzt M. Saliba S. Kapiotis S. Eickhoff P.. (2008). Circulating vascular endothelial growth factor and systemic inflammatory markers in patients with stable and exacerbated chronic obstructive pulmonary disease. Clin. Sci. 115, 225–232. 10.1042/CS20070382 18307413

[B45] Vat S. Haba-Rubio J. Tafti M. Tobback N. Andries D. Heinzer R. (2015). Scoring criteria for portable monitor recordings: a comparison of four hypopnoea definitions in a population-based cohort. Thorax 70, 1047–1053. 10.1136/thoraxjnl-2014-205982 26294685

[B46] Vogelmeier C. F. Criner G. J. Martinez F. J. Anzueto A. Barnes P. J. Bourbeau J.. (2017). Global strategy for the diagnosis, management, and prevention of chronic obstructive lung disease 2017 report. GOLD Executive Summary. Am. J. Resp. Crit. Care Med. 195, 557–582. 10.1164/rccm.201701-0218PP 28128970

[B47] Wang W. Yuan S. Le Grange J. M. Zheng H. Yao T. Peng W.. (2021). Evaluating the performance of five scoring systems for prescreening obstructive sleep apnea-hypopnea syndrome. Sleep Breath. 25, 1685–1692. 10.1007/s11325-020-02227-6 33123926

[B48] Wu Z. Yang D. Ge Z. Yan M. Wu N. Liu Y. (2018). Body mass index of patients with chronic obstructive pulmonary disease is associated with pulmonary function and exacerbations: a retrospective real world research. J. Thorac. Dis. 10, 5086–5099. 10.21037/jtd.2018.08.67 30233884 PMC6129899

[B49] Xiong M. Hu W. Dong M. Wang M. Chen J. Xiong H.. (2019). The screening value Of ESS, SACS, BQ, and SBQ on obstructive sleep apnea in patients with chronic obstructive pulmonary disease. Int. J. Chron. Obstruct. Pulmon. Dis. 14, 2497–2505. 10.2147/COPD.S223354 32009782 PMC6859167

[B50] Young T. Palta M. Dempsey J. Skatrud J. Weber S. Badr S. (1993). The occurrence of sleep-disordered breathing among middle-aged adults. N. Engl. J. Med. 328, 1230–1235. 10.1056/NEJM199304293281704 8464434

[B51] Zhang J. N. Peng B. Zhao T. T. Xiang M. Fu W. Peng Y. (2011). Modification of the epworth sleepiness scale in Central China. Qual. Life Res. 20, 1721–1726. 10.1007/s11136-011-9898-3 21484529 PMC3220816

[B52] Zhang P. Chen B. Lou H. Zhu Y. Chen P. Dong Z.. (2022). Predictors and outcomes of obstructive sleep apnea in patients with chronic obstructive pulmonary disease in China. BMC Pulm. Med. 22, 16. 10.1186/s12890-021-01780-4 34983482 PMC8725359

