# Supplementary material for: A risk-predictive model for obstructive sleep apnea in patients with chronic obstructive pulmonary disease
Source: Front Neurosci. 2023 Mar 17;17:1146424. doi: 10.3389/fnins.2023.1146424 (PMC10065196; doi:10.3389/fnins.2023.1146424)
Supplement: Supplementary file 1 [file Table_1.DOCX]

**Table S1.** **Demographics and clinical characteristics of patients in the training group and validation group**

| **Characteristics** | Training group (n=230) | Validation group (n=100) | *p* value |
| --- | --- | --- | --- |
| OSA | 67 (29.1%) | 29(29.0%) | 0.981 |
| gender |  |  | 0.630 |
| male, n (%) | 193 (83.9%) | 86 (86.0%) |  |
| female, n (%) | 37 (16.1%) | 14 (14.0%) |  |
| age, years | 70.00 (65.00, 77.00) | 70.00 (63.25, 77.00) | 0.982 |
| BMI, kg/m^2^ | 20.30 (17.70, 24.20) | 20.20 (17.63, 24.20) | 0.642 |
| NC, cm | 36 (34, 40) | 36 (34, 38) | 0.741 |
| smoke |  |  | 0.978 |
| Yes, n (%) | 186 (80.9%) | 81 (81.0%) |  |
| No, n (%) | 44 (19.1%) | 19 (19.0%) |  |
| AE in the previous year |  |  | 0.642 |
| Yes, n (%) | 169 (73.5%) | 71 (71.0%) |  |
| No, n (%) | 61 (26.5%) | 29 (29.0%) |  |
| hypertension |  |  | 0.064 |
| Yes, n (%) | 129 (56.1%) | 45 (45.0%) |  |
| No, n (%) | 101 (43.9%) | 55 (55.0%) |  |
| type 2 diabetes |  |  | 0.547 |
| Yes, n (%) | 121 (52.6%) | 49 (49.0%) |  |
| No, n (%) | 109 (47.4%) | 51 (51.0%) |  |
| coronary heart disease |  |  | 0.134 |
| Yes, n (%) | 41 (17.8%) | 25 (25.0%) |  |
| No, n (%) | 189 (82.2%) | 75 (75.0%) |  |
| hyperlipidemia |  |  | 0.260 |
| Yes, n (%) | 111 (48.3%) | 55 (55.0%) |  |
| No, n (%) | 119 (51.7%) | 45 (45.0%) |  |
| routine blood test |  |  |  |
| WBC, × 10^9^/L | 7.02 (5.28, 10.18) | 7.64 (5.38, 10.40) | 0.370 |
| RBC, × 10^12^/L | 4.40 (4.12, 4.81) | 4.35 (4.11, 4.82) | 0.940 |
| Hg, g/L | 134 (123, 141) | 135 (123, 142) | 0.723 |
| HCT, % | 41.6 (38.2, 44.6) | 41.9 (37.3, 44.7) | 0.956 |
| MCV, fl | 92.5 (89.8, 97.9) | 91.7 (88.9, 96.9) | 0.284 |
| MCH, pg | 30.2 (28.8, 31.6) | 30.3 (28.7, 40.0) | 0.577 |
| MCHC, g/L | 323 (313, 332) | 323 (313, 331) | 0.674 |
| coagulation Index |  |  |  |
| D-Dimer, ug/mL | 0.85 (0.42, 2.08) | 0.57 (0.42, 1.56) | 0.197 |
| PT, s | 13.7 (13.3, 14.7) | 13.7 (13.1, 14.9) | 0.685 |
| INR | 1.08 (1.03, 1.25) | 1.10 (1.01, 1.28) | 0.430 |
| APTT, s | 36.1 (32.9, 39.3) | 36.0 (33.0, 39.0) | 0.550 |
| FIB, g/L | 4.20 (3.22, 5.49) | 4.11 (3.12, 5.49) | 0.922 |
| TT, s | 18.2 (16.9, 19.7) | 18.5 (17.3 19.8) | 0.600 |
| blood gas analysis |  |  |  |
| PH | 7.39 (7.32, 7.44) | 7.38 (7.32, 7.44) | 0.879 |
| PaO_2_, mmHg | 77.7 (63.0, 88.5) | 79.0 (64.0, 91.7) | 0.470 |
| PaCO_2_, mmHg | 47.2 (39.0, 66.4) | 46.0 (39.4, 66.4) | 0.631 |
| BNP, pg/mL | 116.4 (56.7, 292.4) | 100.4 (57.6, 352.4) | 0.698 |
| CRP, mg/L | 21.23 (4.89, 53.08) | 21.12 (3.90, 53.08) | 0.521 |
| Questionnaires |  |  |  |
| mMRC, point | 3 (2, 4) | 3 (3, 4) | 0.763 |
| CAT, point | 25.50 (21.00, 31.00) | 26.00 (20.25, 30.00) | 0.740 |
| SACS, point | 4.00 (3.00, 13.00) | 4.00 (3.00, 10.75) | 0.732 |
| mESS, point | 11.00(7.00, 14.00) | 9.00 (6.00, 13.75) | 0.136 |
| PSQI, point | 9 (6, 12) | 9 (6, 12) | 0.944 |
| GOLD Stage, n (%) |  |  | 0.002 |
| 1 | 82 (35.7%) | 33 (33.0%) |  |
| 2 | 65 (28.3%) | 24 (24.0%) |  |
| 3 | 60 (26.0%) | 26 (26.0%) |  |
| 4 | 23 (10.0%) | 17 (17.0%) |  |
| Sleep parameters |  |  |  |
| AHI, times per hr | 5.6 (1.6, 20.8) | 6.2 (1.7, 19.9) | 0.540 |
| ODI, times per hr | 9.7 (1.3, 19.2) | 11.2 (1.5, 20.1) | 0.869 |
| Mean SaO_2_, % | 90.9 ± 2.7 | 91.2 ±1.4 | 0.349 |
| Minimum SaO_2_, % | 74.8 ± 10.6 | 76.2 ± 13.2 | 0.720 |

**Abbreviations:** OSA, obstructive sleep apnea syndrome; BMI, body mass index; NC, neck circumference; AE: acute exacerbation; WBC, white blood cell; RBC, red blood cell; Hb, hemoglobin; HCT, hematocrit; MCV, mean corpuscular volume; MCH, mean corpuscular hemoglobin; MCHC, mean corpuscular hemoglobin concentration; PT, prothrombin time; INR, international normalized ratio; APTT, activated coagulation time of whole blood; FIB, fibrinogen; TT, thrombin time; PH, potential of hydrogen; PaO_2_, arterial oxygen partial pressure; PaCO_2_, arterial carbon dioxide pressure; BNP, brain natriuretic peptide; CRP, C-reactive protein; mMRC, modified Medical Research Council dyspnea scale; CAT, chronic obstructive pulmonary disease assessment test; SACS, sleep apnea clinical scale; mESS, modified Epworth sleepiness scale; PSQI, Pittsburgh Sleep Quality Index; GOLD, Global Initiative for Chronic Obstructive Lung Disease; AHI, apnea hypopnea index; ODI, oxygen desaturation index; SaO_2_, arterial oxygen saturation.
